# Hepatitis C Virus Evasion Mechanisms from Neutralizing Antibodies

**DOI:** 10.3390/v3112280

**Published:** 2011-11-15

**Authors:** Caterina Di Lorenzo, Allan G. N. Angus, Arvind H. Patel

**Affiliations:** MRC - University of Glasgow Centre for Virus Research, Church Street, Glasgow, G11 5JR, UK; E-Mails: caterina.dilorenzo@glasgow.ac.uk (C.D.L.); allan.angus@glasgow.ac.uk (A.G.N.A.)

**Keywords:** hepatitis C virus, neutralizing antibodies, immune evasion

## Abstract

Hepatitis C virus (HCV) represents a major public health problem, affecting 3% of the world’s population. The majority of infected individuals develop chronic hepatitis, which can progress to cirrhosis and hepatocellular carcinoma. To date, a vaccine is not available and current therapy is limited by resistance, adverse effects and high costs. Although it is very well established that cell-mediated immunity is necessary for viral clearance, the importance of host antibodies in clearing HCV infection is being increasingly recognized. Indeed, recent studies indicate that neutralizing antibodies are induced in the early phase of infection by patients who subsequently clear viral infection. Conversely, patients who do not clear the virus develop high titers of neutralizing antibodies during the chronic stage. Surprisingly, these antibodies are not able to control HCV infection. HCV has therefore developed mechanisms to evade immune elimination, allowing it to persist in the majority of infected individuals. A detailed understanding of the mechanisms by which the virus escapes immune surveillance is therefore necessary if novel preventive and therapeutic treatments have to be designed. This review summarizes the current knowledge of the mechanisms used by HCV to evade host neutralizing antibodies.

## 1. Introduction

**Figure 1.**
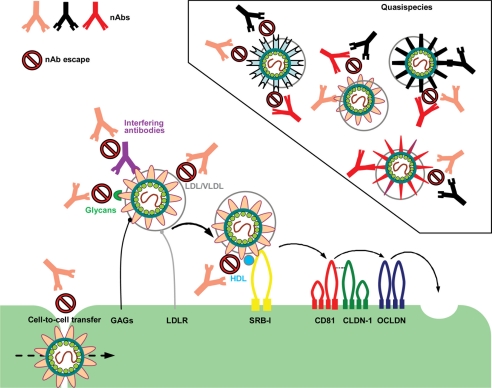
Hepatitis C virus (HCV) entry and escape from nAbs. Several cell surface molecules mediate HCV binding to host cells. GAGs and LDLR may facilitate initial attachment by interacting with HCV glycoproteins and virion-associated lipoproteins, respectively. After the initial binding step, the virion interacts with the entry receptor SR-BI, followed closely by CD81. The tight junction proteins claudin-1 (CLDN-1) and occludin (OCLDN) also contribute to binding, uptake and internalization of HCV by receptor-mediated endocytosis. The failure of nAbs in controlling HCV infection could be caused by several different factors. HCV can rapidly evolve into many quasispecies within an infected individual, therefore outpacing the nAb response; virion-associated lipoproteins and glycans may protect the envelope glycoproteins from nAbs; the presence of interfering Abs could diminish the function of nAbs by preventing them from binding to viral glycoproteins; virus entry may be enhanced by HDL therefore reducing the time window during which the Abs can bind to and neutralize the virus; HCV can infect surrounding cells through direct cell-to-cell contact therefore avoiding being exposed to nAbs. Adapted from Zeisel *et al.* [16] and Angus and Patel [17]. GAG: Glycosaminoglycan; LDL: Low density lipoprotein; VLDL: Very low-density lipoprotein; HDL: High-density lipoprotein; LDLR: Low-density lipoprotein receptor; SR-BI: Scavenger receptor class B; nAb: neutralizing antibody.

Hepatitis C virus (HCV) infection remains a serious burden to public health, affecting 2.0–3.0% of the world’s population [1–3]. HCV is a RNA virus with an enveloped virion belonging to the family Flaviviridae [4]. It contains a positive-sense single stranded RNA genome that is 9,600 nucleotides in length [5]. HCV genomic RNA is composed by a single open-reading frame flanked by 5′ and 3′ non-coding regions. The HCV polyprotein is approximately 3,000 amino acids in length and is cleaved into three structural proteins (core, and envelope glycoproteins E1 and E2) and seven non-structural proteins (p7, NS2, NS3, NS4A, NS4B, NS5A and NS5B). HCV is classified into seven different genotypes that, on average over the complete genome, differ in 30–35% of nucleotide sites [6,7]. Each genotype is further divided into a series of more closely related subtypes that differ in 20–25% of nucleotide sequences [8]. Hepatocytes are the major target cells of HCV. Virus entry into host cells is a complex multistep process involving the presence of several entry factors [9]. Initial host cell attachment involves glycosaminoglycans (GAGs) and the low-density lipoprotein receptor (LDLR), after which the virus appears to interact sequentially with four entry factors: the scavenger receptor class B type I (SR-BI), the tetraspanin CD81 and the tight-junction proteins claudin-1 (CLDN-1) and occludin (OCLDN) [9] (Figure 1).

HCV is a major cause of acute and chronic hepatitis worldwide [10]. Only 20% of infected patients recover spontaneously. The majority progress to chronic infection, which ultimately leads to liver cirrhosis and hepatocellular carcinoma, both leading causes of liver transplantation (LT) [11]. A protective vaccine is not yet available and therapeutic options are still limited. Current standard therapy, which is pegylated interferon-α (IFN-α) combined with ribavirin, is often difficult to tolerate and results in a sustained virological response in only 50% of patients [12]. However, treatment of hepatitis C continues to evolve striving for constant improvement. A large number of new therapies are currently in development, including direct-acting antiviral drugs that target specific HCV enzymes [13,14]. Completed Phase 3 studies of two of these compounds, telaprevir and boceprevir, have provided promising results for patients infected with hepatitis C genotype 1 [14]. Nevertheless, IFN-α and ribavirin were part of all the treatment regimes tested in these studies, thus important adverse effects and several contraindications remain major problems in HCV therapy. Recently, a combination of two direct-acting antiviral drugs, danoprevir and RG7128, has provided hope of developing possible IFN-free treatment regimens [15]. Overall, this treatment was well tolerated and some patients had HCV RNA concentrations below the limit of detection.

Even though several promising tools are in development for the treatment of patients with hepatitis C genotype 1, how useful the novel agents will be in the most difficult to treat patients, such as those with advanced liver disease or after LT, is still unclear. Furthermore, direct-acting antivirals also have to be developed for the other HCV genotypes. In order to improve antiviral therapy and vaccine development, a detailed understanding of the viral and host factors that determine HCV persistence or clearance of infection is essential.

Cellular immune responses are now known to be crucial in controlling HCV infection [18,19]. Spontaneous HCV clearance is associated with a strong, early cellular immune response to multiple HCV epitopes, and both CD4^+^ and CD8^+^ T cell responses are maintained for several years after viral clearance [18,19]. Indeed, failure to mount an effective cell-mediated immune response is associated with the development of chronic infections [18,19]. Conversely, understanding the role that humoral immunity plays during HCV infection has been quite challenging due to the heterogeneity of patient cohorts and HCV strains. Spontaneous resolution without seroconversion has been observed in chimpanzees [20] and humans [21,22]. These studies therefore, support the concept that anti-HCV antibodies are not essential to clear the infection. Nevertheless, there is now some evidence indicating that neutralizing antibodies (nAbs) may play a role in controlling HCV infection. Such antibodies have been reported to emerge during the course of acute HCV infection both in patients [23,24] and in experimentally infected chimpanzees [25]. Furthermore, passive transfer of serum containing HCV antibodies to chimpanzees delayed virus replication upon challenge, suggesting that antibodies can modify the course of infection [26]. Some early studies also suggested an association between the rapid onset of an antibody response to the virus and infection outcome. Patients who spontaneously resolved HCV infection were more likely to have serum antibodies against HCV within the first six months of infection when compared with those who developed persistent viraemia [27,28]. More importantly, a single-source outbreak of HCV provided a unique opportunity to study the impact of nAbs in the control of viral infection [29]. Lack of nAbs in the early phase of infection was associated with the development of chronic HCV despite the induction of cross-neutralizing antibodies in the late phase of infection [29]. Viral clearance was instead associated with a rapid induction of nAbs in the early phase of infection [29]. Nevertheless, it is now known that HCV has evolved several mechanisms to evade host nAbs in order to sustain its own persistence. It is therefore important to gain a better insight into the mechanisms that counteract HCV neutralization in order to facilitate the development of new antiviral treatments and effective vaccines. This review will address the main strategies adopted by HCV to evade host humoral immune responses and how these will impact on the immunotherapeutic potential of nAbs targeting conserved regions of the HCV glycoproteins.

## 2. Viral Evasion Mechanisms from Neutralizing Antibodies

### 2.1. Lipoproteins

Within the serum of infected individuals, HCV exhibits highly heterogeneous density profiles [30–33]. Ultracentrifugation of infected plasma on density gradients revealed an unusually low buoyant density (ranging from <1.06 to >1.25 g/mL) for a small enveloped RNA virus [30–34]. Poorly infectious particles, which were found to be linked to immunoglobulins, were identified at 1.25g/mL whereas highly infectious particles were associated with a density of 1.06mg/mL [35]. The low buoyant density of HCV is due to virus particle association with β-lipoproteins, *i.e.*, very low-density lipoproteins (VLDL) and low-density lipoproteins (LDL) [36,37], including the apolipoproteins B (ApoB) and E (ApoE) [36–43]. For this reason, these low-density HCV particles are referred to as ‘lipo-viro-particles’ (LVP).

HCV particles might directly bind to lipoproteins or incorporate lipoprotein components, such as lipids and apolipoproteins, either through their interaction with the blood of infected patients or through their interaction in virus producer cells [44]. The association of HCV with lipoproteins may facilitate virus entry into target cells. Indeed, the LDL receptor (LDLR) has been shown to internalize HCV associated with LDL and VLDL in various human cell types *in vitro*, leading to infection [38,45,46]. Furthermore, only low-density fractions of infectious serum have been shown to transmit infection to chimpanzees [34] and to infect cultured cells *in vitro* [38,45]. These observations suggest that, lipoproteins associated with the virus are critical for the infectivity of serum HCV and could provide protection against antibody-mediated neutralization, perhaps via shielding of the viral surface glycoproteins. Confirming these observations are data obtained using both HCV/HIV pseudotypic particles (HCVpp) bearing the envelope glycoproteins and cell culture-derived HCV (HCVcc). HCVpp do not associate with lipoproteins thus allowing the investigation of cell entry events specifically linked to the function of E1E2 glycoproteins [42,47,48], whereas HCVcc have a lipid composition resembling that of native HCV [49–51]. Immature intracellular HCVcc virions, which have lower lipoprotein content than released virions, are more sensitive to neutralization by anti-E2 antibodies and less sensitive to anti-ApoE antibodies than released virions [52]. Also, the neutralization of extracellular HCVcc was shown to increase with particle density, suggesting that the efficiency of neutralization is affected by the lipoprotein content of HCV [53]. In line with this theory, a cell culture-adaptive mutation in E2 (I414T) that reduced the lipoprotein content of HCVcc virions also made the virus more sensitive to neutralization by anti-E2 antibodies [52]. Therefore, it seems that the reduced lipoprotein content of the virions resulted in the increased exposure of the glycoproteins, making them more accessible for binding by anti-E2 nAbs. As expected, since HCVpp already lack lipoproteins, the I414T mutation did not alter their sensitivity to anti-E2 nAbs.

**Figure 2.**
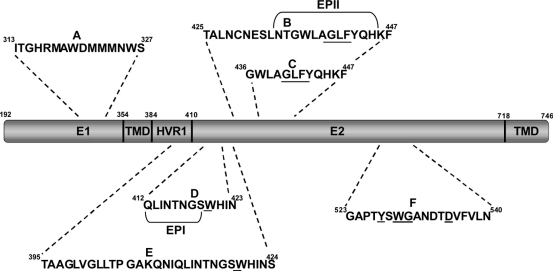
Conserved epitopes recognized by broadly nAbs in E1 and E2. Underlined letters indicate residues critical for E2-CD81 binding. HVR1: Hypervariable region 1; TMD: Transmembrane domain.

Antibody-mediated neutralization of HCV was also shown to be attenuated by high-density-lipoproteins (HDL) present in human serum [54–57]. Evidence to date suggests that HDL stimulates HCV cell entry at a post-binding stage, which reduces the time window whereby nAbs can bind to and neutralize the virus [58]. This process appears to be governed by the hypervariable region 1 (HVR1), located at the N-terminal end of the E2 protein (Figure 2), and also depends on the expression of SR-BI and its selective lipid-uptake function [59]. An essential component of HDL that seems to be responsible for infection enhancement is ApoC-I [56,60]. Anti-ApoC-I antibodies were shown to immunoprecipitate and neutralize HCVcc as well as virus derived from infected chimpanzees, demonstrating that ApoC-I is a component of HCV [42,51,60]. Furthermore, *in vitro* studies have shown that ApoC-I could be transferred from HDL to HCV during SR-BI mediated lipid transfer [60], a mechanism that predisposes the virus envelope for fusion with a target membrane [60]. The role of a serum protein to promote fusion enhancement is another remarkable feature of the ability of HCV to take advantage of blood and lipoprotein components to facilitate its replication.

In summary, lipoproteins may help the virus escape recognition by the immune system and its subsequent neutralization by two main mechanisms: (1) virus association with LDL and VLDL provides protection against antibody neutralization by masking epitopes on viral surface glycoproteins; (2) HDL accelerates viral entry, which limits the exposure of the virus to nAbs.

### 2.2. Glycans

Glycans on viral-derived glycoproteins are produced by the cellular machinery, thus they are often recognized as ‘self’ by the immune system [61–63]. Consequently, glycans associated with viral envelope proteins decrease the immunogenicity of viral particles by shielding important epitopes, thus protecting HCV from Ab neutralization [61,63]. The envelope glycoproteins of HCV are highly glycosylated, typically containing four and 11 *N*-linked glycans in E1 and E2, respectively and have been shown to play a role in protein folding and HCV entry into target cells [64]. More importantly, HCVpp and HCVcc studies have found that at least five glycans on E2 reduce the sensitivity of virions to polyclonal and monoclonal anti-HCV glycoprotein nAbs [65–67], indicating that these glycans function to limit the accessibility of neutralizing epitopes on E2. The reader is directed to an excellent review for a full discussion of this topic [68].

### 2.3. Interfering Antibodies

A novel HCV humoral escape mechanism involving the induction of interfering antibodies has recently been described [69,70]. Broadly nAbs have been found in experimental immune globulin preparations (HCIG) made from anti-HCV positive donors [71]. Nevertheless, the *in vivo* efficacy of HCIG in both chimpanzees and humans has been disappointing [69] and clinical studies have shown that HCIG fail to prevent recurrent infections in patients after LT [72]. Zhang *et al.* hypothesized that non-nAbs present in HCIG could interfere with nAbs in the immune globulin preparation and be responsible for its ineffectiveness. Indeed, the authors identified two epitopes located downstream of HVR1 within the HCV E2 protein, Epitope I (EPI), at amino acids 412–419 and epitope II (EPII) at amino acids 434–446, and showed that EPI, but not EPII, was involved in virus neutralization [69]. Zhang *et al.* put forward the idea that once EPII is bound to an antibody, the site of EPI becomes masked and can no longer be recognized by specific nAbs. Indeed, depletion of antibodies to EPII in plasma from a chronically infected HCV patient and vaccinated chimpanzees, recovered otherwise undetectable, cross-genotype neutralizing activity [70]. These studies provide evidence of a new mechanism by which HCV can escape from antibody responses. If such a mechanism operates *in vivo*, the dynamic interaction between non-neutralizing and neutralizing antibodies may play a key role in the outcome of HCV infection.

### 2.4. Cell-to-Cell Transmission

Many enveloped viruses have evolved mechanisms to allow them to move between cells without diffusing through the extracellular environment. This mode of transmission shields the virus from the innate and adaptive immune effector mechanisms thus facilitating rapid viral dissemination. Examples of cell-to-cell spread can be found in herpesviruses, paramyxoviruses, retroviruses, poxviruses and rhabdovirus [73]. HCV has been recently shown to spread via direct cell-to-cell transfer. Valli *et al.* first reported *in vitro* cell-to-cell spread of HCV from infected human lymphoblastoid B cells to human hepatoma-derived cells [74,75]. Studies using HCVcc also support the notion that virus particles may be transmitted directly between cells [76–78]. Efficient HCV transmission has been reported in the presence of polyclonal and monoclonal anti-HCV glycoprotein antibodies, suggestive of direct cell-to-cell transfer [77,78]. Intracellular virions were shown to remain sensitive to the neutralizing activity of antibodies, confirming that the transmitting viruses were not resistant to the nAbs used [77]. Furthermore, HCVcc chimeras representative of the seven major genotypes [6] were shown to be transmitted by cell-to-cell contact, implying that this route of transmission is common to all viral strains [76].

It is now well established that HCV entry into target cells is dependent on host molecules SRBI, CD81 and the tight-junction proteins CLDN-1 and OCLDN [79–81]. Whether the mechanisms of cell entry during cell-to-cell spread are distinct from those during cell entry by an extracellular virus is unclear. Most notably, there is conflicting data on whether cell-to-cell transmission is CD81 independent [77,78,82] or not [76]. Nevertheless, both CLDN-1 [76] and OCLDN [76,83] have been shown to play a role in cell-to-cell HCV transmission. However, SRBI was shown to have a more prominent role in cell-to-cell transmission, with SRBI-specific antibodies showing preferential inhibition of this infection route [76]. The evidence from these studies support a role for cell-to-cell transfer in nAb evasion and viral persistence *in vivo*.

### 2.5. Quasispecies

A major characteristic of HCV is its genetic heterogeneity. Due to an error-prone replication mechanism and a high viral replication rate, the virus circulates in an infected individual as a population of closely related, yet heterogeneous sequences [84–88]. It has been estimated that the mutation rate of HCV is 1.5–2.0 × 10^−3^ base substitutions per genome site per year [84], which in combination with the production of approximately 10^10^ to 10^12^ virions per day in chronically infected individuals [89], leads to significant generation of viral variants. Collectively, these variants are referred to as quasispecies. Although variations in the viral genome arise initially due to random base substitutions, there is evidence suggesting that humoral immune responses might mediate quasispecies selection, by exerting selective pressure against the predominant strain. This results in the generation of new minor variants that will eventually become more prevalent. Later the immune system will recognize and exert pressure on the new dominant variant and new mutants will be selected [90].

Several studies of HCV sequence evolution in infected chimpanzees support the antibody-mediated immune selection of HCV species. Ray *et al*. studied HCV quasispecies by serially passaging virus obtained during the acute phase of infection through eight chimpanzees [91]. The serial passage experiment was initiated using a well-characterized strain of HCV (H77), obtained from a patient during acute HCV infection in 1977 [92–94]. Weekly assessments for increased levels of alanine aminotransferase allowed identification of the acute phase of infection, and acute-phase serum was used to inoculate the next animal in the series [91]. Plasma containing virus for inoculation of each subsequent animal was collected early during infection in order to minimize the effects of the adaptive immune response on virus evolution. Very little variation was observed in the E2 sequence in each chimpanzee studied. This was a striking finding since substantially greater sequence variation was noted in a cohort of six chronically HCV-infected humans with similar HCV RNA levels who were analyzed in parallel [91]. These findings support the prediction that a quasispecies under reduced selective pressure will undergo reduced change in the predominant sequence. Another study of chimpanzees that developed persistent infection following inoculation with a homogeneous clone found that the early phase of infection was characterized by a high level of viral mutations, suggesting that a high level of immune pressure occurred during this stage. Conversely, during the chronic phase of infection viral mutations decreased suggesting less immune pressure occurring at this later stage [95]. These observations support the idea that mutations accumulate due to selective pressure rather than arising simply as a consequence of long-term HCV replication [95]. In line with this concept, viral mutations were shown to be reduced in hypogammaglobulinemic patients [96].

Various reports have indicated that HVR1 evolves more rapidly *in vivo* than the rest of the viral genome [97,98]. Mutations in HVR1, which would result in escape from the immunosurveillance system, have been suggested to play a major role in the maintenance of persistent HCV infection [99–101]. Analysis of serum samples obtained from patients with chronic hepatitis and from HCV-inoculated chimpanzees showed that HVR1 contains an immunological epitope that is specific for the homologous virus isolate [102–107], and that, in some cases, an HCV with mutated HVR1 could escape recognition by pre-existing anti-HVR1 antibodies [101]. Since HVR1 shows marked sequence variation, and induces antibodies restricted to the homologous viral isolate, it has been suggested that this region functions as a neutralizing epitope [99,101–108]. Indeed, antibodies from infected patients as well as antibodies raised against HVR1 peptides have been shown to bind to HVR1 and inhibit HCV infection [101,105]. Although various studies have hypothesized that the sequence variability of virus species, particularly in HVR1, provides an indicator of progression to chronicity [99–101,109], persistent infections could still be established in the absence of the HVR1 domain both *in vivo* and *in vitro* [110,111]. Despite these delta HVR1 viruses having reduced infectivity levels, it is clear from these studies that this region of E2 is not critical for virus entry or release. The reduced infectivity of delta HVR1 viruses is in agreement with a role of HVR1 in the binding of E2 to the SRBI receptor [112]. However, since deletion of HVR1 does not completely abrogate HCV infectivity it is possible that other virion components, such as the LDLs and VLDLs [59], are involved in SRB1 binding. Furthermore, it was demonstrated that both *in vitro*- and *in vivo*-derived HVR1-deleted HCVcc had greatly increased neutralization susceptibility to polyclonal and monoclonal anti-HCV glycoprotein antibodies [110,113]. These findings suggest that in the absence of HVR1 the virus exposes key nAb epitopes, thus facilitating effective neutralization. Therefore, HVR1 has been suggested to function as an immunologic decoy during infection by masking a deeper, more highly conserved structure within the viral envelope [109,110,114].

In addition to HVR1, nAbs escape mutations have also been documented in other regions of the E2 glycoprotein. We and others have reported the *in vitro* selection of neutralization resistant viruses by propagating HCVcc in the presence of inhibitory concentrations of antibodies targeting conserved regions of the E2 glycoprotein [115–117] (Figure 2, Table 1). In each study, single amino acid changes within the epitope of each antibody were responsible for the viruses’ neutralization resistant phenotype (Figure 2, Table 1). Mutations within the conserved E2 neutralizing epitopes have also been identified *in vivo*. In a recent study, the viral glycoprotein sequences isolated from a chronically infected patient over a 26-year period were used to characterize the neutralization potential and binding affinity of a panel of anti-HCV E2 human monoclonal antibodies (mAbs) [118]. This study revealed multiple escape variants to the various antibodies tested with one particular isolate (02.E10) containing mutations located outside the CD81 binding site. HCVpp bearing this glycoprotein sequence had reduced binding affinities for all human antibodies tested and for CD81 and showed reduced infectivity. Surprisingly, this isolate was also resistant to inhibition by the mouse monoclonal nAb AP33, despite containing no mutations within the conserved linear epitope of this antibody (Figure 2). Therefore, it appears that the mutations located outside the receptor binding sites resulted in structural changes leading to complete escape from nAbs. However, another consequence of these mutations was reduced virus infectivity due to lower E2-CD81 binding [118]. More recently Duan *et al.* reported another antibody escape mutation (Q412H) in chronic HCV carriers that was located within EPI [119]. While EPI-specific antibodies neutralized HCVcc *in vitro*, they did not neutralize HCVcc containing the Q412H mutation [119]. More importantly, plasma obtained from a chimpanzee that had anti-E1/E2 antibodies following experimental immunization, neutralized the wild-type HCVcc but failed to neutralize the mutant virus, suggesting that the Q412H mutation found in naturally occurring variants could indeed represent an antibody escape mutation [119].

## 3. Implications for Passive Immunotherapy

The aforementioned nAb escape mechanisms employed by HCV may partly explain the limited success of clinical trials using polyclonal and monoclonal antibodies (mAbs) for passive immunotherapy. As mentioned earlier, a phase II clinical trial using high doses (75–200 mg/kg for 14 weeks) of an HCIG, failed to suppress HCV RNA levels or prevent recurrent infection in LT patients [72]. Preparations of broadly neutralizing mAbs are better alternatives for immunotherapy as they lack many of the problems associated with HCIG products, including interfering antibodies and unknown nAb concentrations as well as a number of manufacturing and safety issues. To date, only one human mAb (HCV-AB68) has been clinically evaluated for preventing HCV infection. Administering high doses (20–240 mg) of HCV-AB68 to chronically infected patients during and after LT, failed to prevent graft reinfection. Reductions in HCV RNA levels were observed in patients who received higher doses (120–240 mg) on day 2 post-LT, however, these effects were lost after day 7 when the dosing frequency was decreased [131]. More recently, HCV-AB68 was used in Phase 1B clinical trials in patients with chronic hepatitis C by administering single and multiple doses of up to 120 mg [132]. At the highest dose, transient decreases in HCV RNA levels occurred immediately following infusion, which rebounded to baseline levels within 24–48 hours. HCV-AB68 clearly demonstrates some neutralizing activity *in vivo* and it has been suggested that more frequent daily dosing at higher concentrations may improve the treatment response. Although, it has been demonstrated that HCV-AB68 has high-affinity HCV neutralizing properties against genotype 1b HCVpp, its cross-reactivity towards other genotypes is unknown [133]. Furthermore, the E2 epitope targeted by HCV-AB68 has not been fully mapped and therefore may contain variable regions in E2. Therefore, the presence of HCV-AB68 escape variants within the infected patient’s quasispecies population could explain the transient reductions in viraemia observed in these studies.

The selection of neutralization escape variants is an underlying risk of monotherapy using nAbs targeting the HCV glycoproteins. However, recent studies suggest that certain broadly nAbs are less prone to the selection of viral escape variants. The strongest evidence comes from the AR3A and AR3B conformational nAbs, which at high concentrations (200 mg/kg), could completely protect a human liver chimeric mouse model from challenge with a patient-derived heterologous HCV quasispecies swarm [128]. Using the chimpanzee model, the nAb HCV-1 [134] was also shown to protect against viral challenge at a 250 mg/kg dosage. Despite the high conservation of the E2 linear epitope targeted by HCV-1 [121], HCVcc escape mutations have been identified against antibodies targeting this region (Table 1). These mutations also altered the glycoprotein conformation, which either impaired virus entry [116] or increased the sensitivity of virions to inhibition by other anti-glycoprotein nAbs [115]. Viruses with either phenotype are not likely to maintain a persistent infection *in vivo*. However, it is important to note that the phenotypes of such escape variants may not accurately reflect their *in vivo* behavior. Indeed, there have been numerous reports describing inconsistencies between the replication properties of infectious HCV isolates *in vitro* and *in vivo* (reviewed in [135]). Therefore, it is questionable whether the *in vivo* fitness of these escape variants would be compromised.

Very recently, HCVcc escape experiments have been performed using the conformational anti-E2 nAbs CBH-2, HC-1 and HC-11 (Table 1) [117]. The replication properties of CBH-2 escape variants were similar to the parental virus, whereas the HC-11 escape variants were severely impaired as a result of the mutations reducing virus binding to the CD81 co-receptor. Interestingly, HCVcc was unable to escape from nAb HC-1 and at critical antibody concentrations complete virus deterioration occurred. It is believed that each contact residue for HC-1 is essential and the induction of escape within this epitope leads to a deleterious change in virus function or structure. Therefore, antibodies targeting this epitope alone such as HC-1 are of great therapeutic value given the required integrity of their E2 epitope for virus proliferation. Despite the wealth of information provided by cell culture and animal model studies on these nAbs, it is difficult to estimate what concentrations of antibody would be required to offer adequate protection against graft re-infection post-LT. Using blends of high-affinity broadly nAbs each recognizing different and non-overlapping epitopes on E1 and E2 may be required to tackle the high variance of HCV *in vivo* along with its numerous other nAb shielding mechanisms (Figure 1). Also, increasing the affinity of broadly nAbs to virus particles would lessen the effect of the escape mechanisms involving lipoproteins and glycans. In a very recent study the potency of the HC-1 nAb was further enhanced by affinity maturation [136]. The affinity matured HC-1 IgG clones showed markedly improved E2 binding and viral neutralization. Remarkably, an HCV isolate not neutralized by wild-type HC-1 was neutralized by affinity-matured HC-1 IgG clones. In the LT setting, highly potent nAbs such as HC-1 may reduce the likelihood of graft reinfection, hence concomitantly reducing nAb-resistant viral spread occurring through the cell-to-cell route. Cell-to-cell transfer is arguably the greatest hurdle to tackle in terms of immunotherapy, particularly during chronic infection. Most of the glycoprotein-specific nAbs tested, including HC-1 have proven ineffective against this mode of transfer [76–78]. However, Brimacombe and co-workers observed two anti-E2 antibodies (9/27 and 11/20) that could reduce cell-to-cell transmission, indicating that this mode of transfer does not occur across a sealed cellular junction but instead may utilize a mechanism similar to the antibody permeable virological synapse of HIV [137,138]. The epitopes of 9/27 and 11/20 are located within variable regions of E2 within the 1a genotype of HCV making such nAbs impractical for immunotherapy. Many of the broadly nAbs listed in Table 1 have yet to be tested for inhibition of cell-to-cell transmission. Therefore, there remains the exciting possibility that cross-reactive nAbs exist with the ability to neutralize virus infection occurring via the cell-free and cell-to-cell route. Overall, major progress has been made in understanding the antibody mediated neutralization of HCV, which will hopefully contribute to the development of successful immunotherapeutic regimes.

## Figures and Tables

**Table 1.** Broadly neutralizing anti-E1 and -E2 antibodies. Letters A to F indicate the antibody epitopes shown in Figure 2.

**Antibody**	**Epitope**	**Escape Mutation(s)**
IGH505 [120]	A (Linear)	-
IGH526 [120]	A (Linear)	-
95-2 [121]	D (Linear)	-
HCV-1 [121]	D (Linear)	-
AP33 [122–124]	D (Linear)	N415Y^§^, N415D*, N417S*, G418D*
3/11 [124]	D (Linear)	N415Y^§^, N415D*, N417S*, G418D*
CBH-5 [117,125,126]	F (Conformational)	-
A8 [127]	F (Conformational)	-
1:7 [127]	F (Conformational)	-
AR3A-D [128]	E, C, F (Conformational)	-
e137 [129]	D, F (Conformational)	-
e20 [130]	F (Conformational)	-
CBH-2 [117,125,126]	B, F (Conformational)	D431G^†^, A439E^†^
HC-1 [117,118]	F (Conformational)	No escape
HC-11 [117,118]	B, F (Conformational)	L438F^§^

Symbols indicate the effect of each escape mutation on virus fitness:

§ Impaired viral spread;

* Increased sensitivity to nAbs;

† Wild-type phenotype;

- not done.
